# Use of sintilimab in primary adenosquamous carcinoma of the liver results in pathological complete response: a case report and literature review

**DOI:** 10.3389/fimmu.2025.1578368

**Published:** 2025-04-30

**Authors:** Zhiqing Bai, Yu-Ann Chen, Ying Xiao, Jianping Song, Jianwei Song, Canhong Xiang

**Affiliations:** ^1^ Hepatopancreatobiliary Center, Beijing Tsinghua Changgung Hospital, School of Clinical Medicine, Tsinghua Medicine, Tsinghua University, Beijing, China; ^2^ Department of Pathology, Beijing Tsinghua Changgung Hospital, School of Clinical Medicine, Tsinghua Medicine, Tsinghua University, Beijing, China; ^3^ Department of General Surgery, Lhasa People’s Hospital, Lhasa, China

**Keywords:** adenosquamous carcinoma of liver, immune checkpoint inhibitors, pathological complete response, sintilimab, PD-1

## Abstract

Adenosquamous cell carcinoma (ASC) is a rare and aggressive malignant tumor which consists of both adenocarcinoma (AC) and squamous cell carcinoma (SCC) component types. Although ASC can sometimes develop in the stomach, pancreas, gallbladder and thyroid, it rarely occurs in the liver. As such, primary ASC of the liver remains a poorly understood malignancy due to both the paucity of reported cases and scarcity of available published data. As such, while the use of immune checkpoint inhibitors (ICIs), including PD-1 and PD-L1 antagonists, has profoundly changed the treatment paradigm and outcomes in most tumors, there is virtually no previous documentation for the application of ICIs in the treatment of primary hepatic adenosquamous carcinoma. Herein, we report a clinical case of a 54-year-old woman with metachronous double primary tumors, one of which was dMMR ASC of the liver and received 8 cycles of single-agent immunotherapy using sintilimab. The post-treatment response was evaluated as a pathological complete response (pCR).

## 1 Introduction

Adenosquamous cell carcinoma (ASC) of the liver is a rare variant of cholangiocarcinoma and an even less commonly encountered malignancy (1, 2). Owing to the paucity of ASC of the liver, the diagnosis and treatment of this rare tumor remains elusive, the treatment response using immunotherapy has previously never been documented in the literature (3). Here, we report a case of a 54-year-old female whose diagnosis of ASC of the liver was confirmed through biopsy pathology and whose immunohistochemistry reveals a mismatch repair deficiency (dMMR). After receiving 8 cycles of single-agent immunotherapy, the patient underwent laparoscopic partial hepatectomy, the postoperative pathology of which indicated a pathological complete response (pCR).

## 2 Case report

The patient is a 54-year-old female who was admitted to the hospital due to the presence of a liver mass that had been discovered six months prior. In reviewing the patient family history, it was found that the patient’s father had passed away due to colon cancer. In addition, the patient was previously diagnosed with atypical endometrial hyperplasia in 2021, due to having an increased menstrual flow which led to receiving a curettage biopsy at another hospital. On August 8^th^ 2021, the patient underwent a radical hysterectomy for uterine cancer, which included a complete removal of the uterus and its accessory organs. Pathological findings confirmed a moderately differentiated endometrioid carcinoma of the uterus, FIGO Grade II. Immunohistochemistry (IHC) showed negative results for MLH1 and PMS2, and positive results for MSH2 and MSH6, indicating a mismatch repair deficiency (dMMR) in the tumor tissue. The patient was then discharged after recovery and has since underwent regular follow-up exams post-surgery.

**Figure 1:**
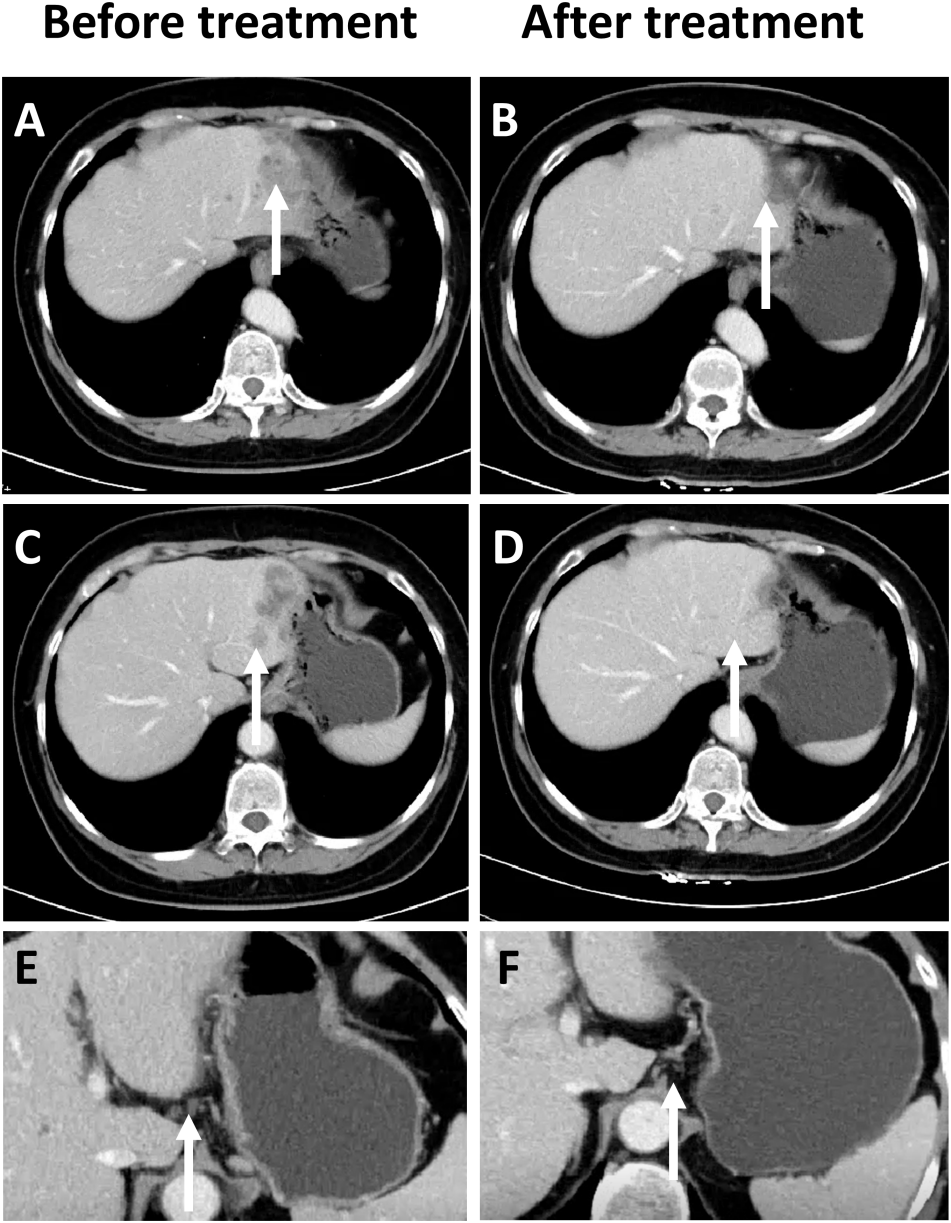
Comparison of CT Imaging changes, prior to and following immunotherapy in patients. **(A)** Prior to treatment, a low-density mass can be observed in the left lobe of the liver at the site indicated by the arrow (white). **(B)** After treatment, tumor regression can be observed in the left lobe of the liver. **(C)** Prior to treatment, satellite lesions can be observed at the edge of the main tumor in the left lobe of the liver. **(D)** After treatment, the satellite lesions have completely regressed. **(E)** Enlarged lymph nodes can be observed in the hepatogastric ligament. **(F)** The size of the previously enlarged lymph nodes are completely reduced.

During a routine follow-up in January 2023, elevated CA19–9 levels (276.9 U/ml) along with abdominal distension was noted. An abdominal CT scan (as shown in **Figure 1**) revealed an irregular low-density lesion in the left liver, measuring approximately 47mm×33mm×27mm with multiple satellite lesions, and lymph node metastasis in the hepatogastric ligament which could not be ruled out (**Figures 1A, C, E**). The outpatient multidisciplinary team (MDT), including experts from oncology, hepatobiliary and pancreatic surgery, and pathology, recommended a liver biopsy be performed so as to clarify the origin of the liver mass along with additional immunohistochemical testing to determine its MMR status.

**Figure 2:**
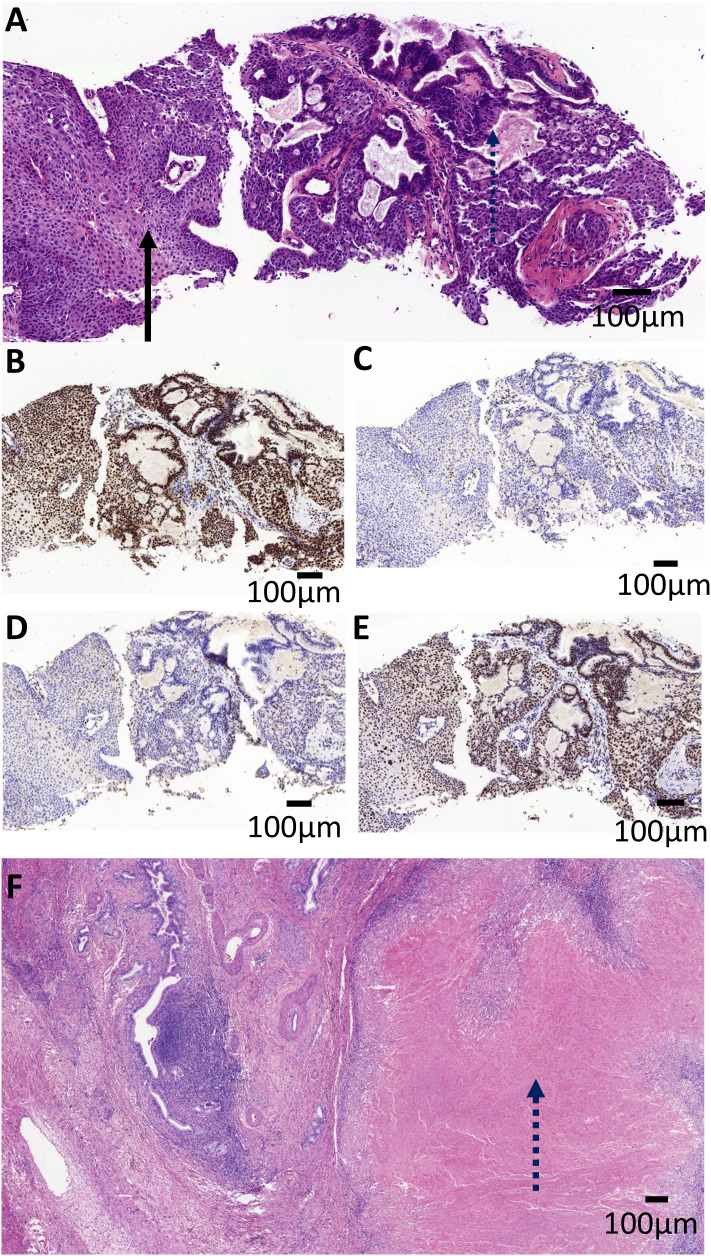
Pathological imaging of the patient tumor. **(A)** The pre-operative biopsy revealed the tumor was composed of adenocarcinoma with tubular pattern (the dashed arrow) and squamous cell carcinoma with nest architecture (the solid arrow). The tumor cells were positive for MLH1 **(B)** and PMS2 **(E)**, and negative for MLH2 **(C)** and MSH6 **(D)**. **(F)** Microscopic examination of the post-operative specimens revealed diffuse and complete necrosis of the neoplasm (the dashed arrow), indicating a pathological complete response (pCR). Scale bar, 100 mm.

On March 15^th^ 2023, an ultrasound-guided liver mass biopsy was performed, and the pathology confirmed a diagnosis of ASC of the liver. Immunohistochemistry results showed: P40 (+), CK19 (+), CK7 (+), Ki67 (40%), P53(mutant expression), MLH1 (+), MSH2 (-), MSH6 (-), PMS2 (+) (**Figures 2A–E**). Given the patient’s family history of colorectal cancer, the presence of two metachronous primary tumors (endometrial carcinoma and hepatic ASC), and immunohistochemical evidence of mismatch repair deficiency in both tumors, the multidisciplinary team (MDT) made a clinical inference of Lynch syndrome. However, due to financial reasons, the patient refused further genetic testing. Given the presence of multifocal lesions and enlarged lymph nodes indicating a high risk of recurrence following surgery, the dMMR status, and likelihood of Lynch Syndrome, the MDT experts concurred that immunotherapy would be the patient’s best treatment choice. The recommended treatment plan included 200 mg of sintilimab every 3 weeks intravenously and 8 mg of lenvatinib every day orally. Sintilimab is an engineered PD-1 inhibitor which has shown greater PD-1 binding affinity *in vitro* than either nivolumab and pembrolizumab, and superior PD-1 occupancy and antitumor effects in humanized mouse models (4). Furthermore, it has exhibited efficacy in both adenocarcinoma and squamous cell carcinoma pathological types and therapeutic potential in a wide range of malignant liver and biliary tract tumors (5, 6). Treatment using Sintilimab was ultimately chosen based on several factors such as its relatively lower cost compared to other ICIs and its demonstrated efficacy in cases of biliary tract malignancies (7, 8). However, due to gastric discomfort in the first week of neoadjuvant therapy, oral lenvatinib use was discontinued. Ultimately, the patient only received 8 cycles of single-agent sintilimab neoadjuvant therapy prior to surgery. Post-treatment, serum CA19–9 levels decreased from 276.9 U/ml to 26.34 U/ml (normal range). As shown in **Figures 1A**, **C**, a low-density shadow and satellite lesions can be observed in the left lateral lobe of the liver prior to treatment. Both the tumor lesions and satellite lesions had almost completely disappeared after the neoadjuvant therapy (**Figures 1B**, **D**). Even the previously suspected metastatic lymph nodes in the hepatogastric ligament showed significant shrinkage in comparison to prior (**Figures 1E, F**). Therefore, the patient underwent laparoscopic partial liver resection and regional lymph node dissection. As shown in **Figure 2F**, the postoperative pathology results revealed diffuse and complete necrosis of the neoplasm, indicating a pathological complete response (pCR) to treatment. A 16-month follow up found no tumor recurrence. The patient’s clinical workup, including medical history, diagnostic and therapeutic procedures, and follow-up, is illustrated in **Supplementary Figure 1**.

## 3 Discussion

The current consensus dictates that ASC of the liver is a rare variant of intrahepatic cholangiocarcinoma (9). It was reported by the National Cancer Center Hospital in Japan that from 2016 to 2017, the incidence of rare intrahepatic bile duct tumors was 0.736%. ASC of the liver accounted for 4.96% (30/605) of rare intrahepatic bile duct tumors (10). Likewise, according to the SEER (Surveillance Epidemiology and End Results) database, the average annual incidence of ASC in the United States was only 1.77/100,000 from 1973 to 2015. Among these, the total cases of ASC of liver was 18 or 0.6% of all cholangiocarcinoma cases, thus confirming the rarity of primary ASC of liver occurrence on a multinational scale (11).

While most published reports have been presented as either singular case reports or a series of small scale studies, we have provided detailed tables consisting of comprehensive patient information obtained from case reports conducted in the last 30 years (**Table 1**) and which also summarize the results of a series of small case studies (**Table 2**).

**Table 1:** A summary of reported cases of ASC of liver of the past 30 years.

Authors	Tumor marker	Symptoms	Localization/Size (cm)	Radiologic diagnosis	Pathological characteristics	Treatment	Outcome
Takahashi H 1997 (12)	CEA 43.2ng/ml; CA19-9 2300U/ml	Fever; Jaundice	Medial and anterior segments/8cm	Liver abscess	Lymph node metastasis	Right trisegmentectomy	3 months
Sasaki H (13)	CA19-9 950U/ml	Epigastric discomfort	Medial and lateral segments/6cm	Low-density mass	Satellite nodes; Lymph node metastasis	Left hepatectomy	15 months
Liu Y M (14)	CA19-9 97.02U/ml	Abdominal pain	Right lobe/7.2cm	Low-density mass	/	Right hepatectomy	>10 months
Zhou S Y (15)	CEA 16.75ng/ml; CA19-9 876.40U/ml	No	Left lobe	Left hepatic duct	No lymph node metastasis	Left hepatectomy	21 months
Gao S (16)	CEA 6.9ng/ml; CA19-9–200 U/ml	Anorexia; fatigue	Right lobe/8cm	Rim enhanced	/	Right hepatectomy	10 months
Park S Y (2)	CA19-9 994.9U/ml	Abdominal discomfort	Right lobe/8cm	Rim enhanced	No lymph node metastasis	Right trisegmentectomy	/
Watanabe Y (17)	SCC 18.6 ng/ml; CEA 4 ng/ml	Fever	Left lobe/6cm	Low-density mass	Diaphragmatic and pericardial; Lymph node metastasis	HAIC+laparoscopic hepatectomy	>6 months
Hayashi T (18)	CA19-9–199 U/ml; SCC 6.6 ng/ml	Fever, vomit	Left lobe/5cm	Low-density mass	/	/	2 months
Nosaka T (19)	CA19-9–58 U/ml; SCC 7.6 ng/ml; CYFRA24.5ng/ml; NSE 63.6ng/ml	Abdominal pain	Right lobe/10cm	Rim enhanced	Celiac lymph nodes metastasis; Right lung metastasis	Chemotherapy+HAIC	14 months
Suzuki E (20)	CEA 52.31ng/ml; CA19-9 4785.0U/ml	Fever	Left lobe/8cm	Low-density mass	No lymph node metastasis	Laparoscopic hepatectomy +HAIC	17 months
Nam K H (1)	CEA 13.6ng/ml; CA199 433.9U/ml	Epigastric pain	Left lobe/6.5cm	Rim enhanced	Lymph node metastasis	Left hepatectomy	8 months recurrence
Kwon OS (21)	CEA 20.8 ng/ml; CA19-9 95.9 U/ml	Fever	Left lobe/6cm	Rim enhanced Low-density mass	No lymph node Metastasis; Satellite nodules; Right diaphragm invaded	Left hepatectomy + chemotherapy	>8 months
Yeung JT (22)	CA19-9–130 U/ml.	Epigastric pain	Left lobe	Rim enhanced low-density mass	Enlarged pericardial lymph node	/	/
Kang GH (23)	CEA 7.2 ng/ml; CA19-9–1600 U/ml	Fever	Left lobe/5cm	Rim enhanced Low-density mass	Hepatoduodenal ligament; No. 8 metastasis	Left hepatectomy	>15 months
Harino T (24)	CEA 8.0 ng/ml; CA19-9 19,196 U/ml	Hepatolithiasis	Left lobe/3cm	Hepatolithiasis	No lymph node metastasis	Left hepatectomy + chemotherapy	>11 months
Wei D (25)	CA-125 65.82 ng/ml; CA19-9 361.6 U/ml; SCC elevated	Abdominal pain Fever	Left lobe/7cm	Low-density mass	Necrosis	Laparoscopic hepatectomy	>2 years
Daiku K (26)	CA19-9–417 U/ml	PSC	Right liver lobe/4.6cm	Low-density mass	No lymph node metastasis	Right hepatectomy	>4 years
Wu PH (27)	CEA, AFP normal	Intermittent epigastralgia	Right liver lobe/9.5cm	Low-density mass	/	Laparoscopic right hepatectomy	/
Nakai T (28)	SCC 42.5 ng/ml	Abdominal pain	Left and right liver lobe/	Low-density mass	Diaphragm invasion; Inferior vena cava invasion	Partial hepatectomy +HAIC	>12 months
Demir G (29)	/	Abdominal pain	Right liver lobe/5cm	Solid mass	/	Hepatectomy + chemotherapy	>8 years
Shimizu S (30)	CEA 5.2 ng/ml; CA19-9–285 U/ml	Epigastralgia	Left liver lobe/4cm	Left and right liver lobe/	Left liver:ASC Caudate: HCC	Left hepatectomy with caudate lobectomy	/
Yokota H (31)	CA-125–588 ng/ml; CEA 16.3 ng/ml; CA19-9–459 U/ml; SCC normal	Fever Epigastralgia	Middle liver	Rim enhanced Low-density mass	/	Not excisable Radiation+HAIC	13 months
Yamao K (32)	CEA 6.1 ng/ml; CA19-9–9290 U/ml; SCC 5.1 ng/ml;	PSC history; fever and epigastric pain	Left liver lobe/6.2cm	Rim enhanced Low-density mass	/	Left hepatectomy	2 months

CEA, carcinoembryonic antigen; CA19-9, carbohydrate antigen 19-9; CA-125, carbohydrate antigen CA-125; SCC, squamous cell carcinoma antigen; PSC, primary sclerosing cholangitis; HCC, hepatocellular carcinoma; HAIC, hepatic arterial infusion chemotherapy.

**Table 2:** A summary of a series of small case studies of ASC of the liver.

Authors	Patients Numbers	Age(y)	Sex(M:F)	Symptoms	Tumor marker	Pathologic characteristics	Medial survival (months)	Relative Risk of Death
Kobayashi M (33)	30	/	20:10	Absent:Present: 15:15	/	Intrahepatic metastasis:8 Lymph node metastasis:14	8.0	Lymph node metastasis Total bilirubin (Surgery)
Sasaki H (13)	36	62	25:11	Abdominal pain 68%, Fever 48%, Loss of weight 40%.	/	/	8.7(operated) 2.2(not operated)	/
Takahashi H (12)	8	60.6	6:2	Absent:Present: 1:7	/	Intrahepatic metastases:37.5(3/8) Lymph node metastasis:88%(7/8)	5.1	/
Gou Q (3)	15	63.6	12:3	Absent:Present: 15:0	CA19–9 elevated 100%(15/15) CEA elevated 80%(12/15)	Intrahepatic metastases 7%(1/15) Lymph node metastasis 73%(8/15)	7(operated) 3(not operated)	Lymph node metastasis
Yeh CN (9)	10	58.9	7:3	Abdominal pain 75% Loss of weight 41.7% Fever 25%	CA19–9 elevated 47.4% CEA elevated 20%	Associated hepatolithiasis 60%(6/10)	5.7(operated)	/
Isa T (34)	4	70.3	3:1	Abdominal pain 50%	/	Lymphatic involvement 50%(2/4) Mass forming type 75% (3/4) Periductal infiltrating typr 25%(1/4)	/	/

Clinicopathological characteristics: Based on the summary of the above literature, clinicopathological information profiling of patients with ASC of the liver reveals a higher incidence in males and that the most commonly presented symptoms are upper abdominal discomfort, fever, and other (3, 9, 12, 13, 33, 34). However, it is difficult to distinguish ASC of the liver from other primary liver tumors like hepatocellular carcinoma (HCC) or intrahepatic cholangiocarcinoma (ICC) solely through analysis of clinical symptoms, laboratory tests, and imaging examinations. Similarly, due to the limited sample size, the reported elevated levels of CA19–9 and CEA in ASC of the liver patients tend to vary across different studies. In spite of this, it was found that the elevation rate of CA19–9 in ASC of the liver patients to be relatively high, based on series of small case studies by various researchers such as Gou Q (3), and Yeh CN (9). Although this seems promising, CA19–9 alone cannot distinguish ASC of the liver from ICC. A few other reports have indicated elevated SCC antigen levels in patients with ASC of the liver (18, 19, 28, 32). SCC antigen can be theoretically used as a potential marker to distinguish ASC of liver from HCC/ICC. However, whether or not low detection rate of SCC antigen is due to its inherently low levels in ASC patients or is simply due to insufficient testing remains inconclusive. In other words, it is highly recommended that SCC antigens are simultaneously tested to further support differential diagnosis.

Pathogenesis and treatment strategies: A few studies have reported that ASC of the liver can be associated with conditions such as primary sclerosing cholangitis (PSC) (26), and hepatolithiasis (24). As shown in **Table 1**, ASC of the liver typically has a tumor diameter which exceeds 5 cm and imaging results including rim-enhanced, low-density masses sometimes accompanied by satellite lesions (13, 21), regional (12, 13, 17) and distant (17, 22, 28) lymph node metastasis, and invasion of adjacent organs or distant metastases (19, 21, 28). Since ASC of the liver is a rare condition, there is a limited understanding of its pathogenesis and optimal treatment strategies. The prognosis of ASC of the liver is commonly known to be poor and surgery is the preferred treatment. Sasaki et al. (13) reported a median survival of 8.7 months for patients who underwent surgery and 2.2 months for those who did not. The basic surgical principles for ASC should be similar to the treatment algorithm for ICC given their similar rapid proliferation and high invasiveness. The best treatment strategy for most ASC patients should be established by a multidisciplinary team. Similarly to ICC, the first step is assessment of tumor resectability, typically evaluated using CT and/or MRI with MRCP. PET and/or EUS-guided fine-needle aspiration/biopsy is often necessary to confirm or exclude metastasis, given the high incidence of lymph node metastases (35). Surgical resection is preferred specifically when there is only one tumor and no regional lymph node metastases are present. In addition, regional lymphadenectomy should be a standard procedure during liver resection (36).

Similarly to ICC, if regional LNM or multiple tumors are present in ASC patients, the choice between resection and drug therapy should depend on the extent of metastasis and the number of tumors. In spite of this, systemic and local treatment effects for ASC of the liver remain uncertain. Nosaka T (19). reported a case of ASC of the liver with distant metastasis that initially responded to gemcitabine-cisplatin therapy, but tumor marker levels rebounded once again after only six months. Hepatic arterial infusion chemotherapy (HAIC) with cisplatin (CDDP) and 5-fluorouracil (5-FU) was then administered, leading to tumor shrinkage. The patient ultimately died 14 months following initial treatment. Demir G (29) reported an ASC patient who underwent segmentectomy followed by cisplatin-based chemotherapy and survived for more than eight years. Suzuki E (20) described another ASC patient who experienced rapid postoperative recurrence with multiple metastases within three months. This patient’s disease was controlled with cisplatin and 5-fluorouracil and passed away 14 months after surgery. Reports by Nakai T (28), Yokota H (31) also demonstrated that HAIC could effectively control tumor progression and improve patients’ quality of life. Based on these case reports, which provide detailed descriptions of treatment strategies and outcomes, chemotherapy or HAIC are also potentially effective treatment options for ASC of the liver. However, past treatment strategies for ASC of the liver have predominantly involved systemic chemotherapy or local therapies such as HAIC and radiotherapy, while the efficacy of targeted therapy and immunotherapy has been rarely reported in the literature. Sintilimab, a type of ICIs, has demonstrated significant therapeutic potential in the management of malignant hepatobiliary tumors (37–40). The therapeutic effect of sintilimab in this case reveals the potential of ICIs in the treatment of ASC of the liver. In the management of ASC of the liver with immune checkpoint inhibitors (ICIs), it is essential to monitor not only treatment efficacy but also the occurrence of immune-related adverse events (irAEs), including immune-mediated pneumonitis, colitis, and hepatitis. As far as we know, this is the first reported case in which one of the two primary malignancies of the patient is ASC of the liver. This is also the first report on the application of ICIs in primary ASC of the liver, although there are some recent studies which show that ICIs can achieve good results in metastatic hepatic ASC (41, 42). The precise mechanism underlying the response of hepatic ASC to immune checkpoint inhibitors (ICIs) remains to be elucidated—particularly whether such efficacy is driven by tumor-intrinsic mismatch repair deficiency or a germline predisposition such as Lynch syndrome. While sporadic case reports have described adenosquamous carcinoma in patients with Lynch syndrome (43, 44), there is currently no established evidence indicating a common association between ASC and Lynch syndrome. To optimize treatment strategies, prospective studies are essential to identify predictive biomarkers and define which patients are most likely to benefit from immunotherapy. In addition, Hong et al. (45) has recently published a case report of a woman with ASC of the extrahepatic biliary tract with multiple lymph node metastases which is characterized by HER-2 amplification. This patient received a chemotherapy regimen consisting of gemcitabine, cisplatin, and trastuzumab, displaying a progression-free survival (PFS) of 5 months. Gou et al. reported an inoperable ASC patient with intrahepatic metastasis who received sorafenib-targeted therapy and survived for 9 months (3).

In conclusion, ASC patients may benefit greatly from systemic chemotherapy, HAIC therapy, targeted and immune therapy. We should make greater efforts to explore these treatment approaches in order to improve patients’ quality of life or extend their survival in order to further develop the most optimal treatment of ASC of the liver.

## 4 Conclusions

This case presents the first reported instance of a patient with primary hepatic adenosquamous carcinoma (ASC) that achieved a pathological complete response (pCR) following immunotherapy. This outcome highlights the potential of immune checkpoint inhibitor use in treating ASC of the liver, particularly in tumors with dMMR status, providing many valuable insights for the future management of this rare malignancy.

## Data Availability

The original contributions presented in the study are included in the article/**Supplementary Material**. Further inquiries can be directed to the corresponding author.

## Supplementary material

Supplementary Figure 1 Timeline of treatment and follow-up. Clinical timeline of diagnosis, treatment, and follow-up. The patient underwent a radical hysterectomy for uterine cancer in August 2021. In January 2023, a low-density liver lesion was detected during routine follow-up. A liver mass biopsy in March 2023 confirmed adenosquamous carcinoma (ASC) of the liver. The patient received eight cycles of sintilimab as neoadjuvant immunotherapy from March to October 2023, followed by laparoscopic partial liver resection. As of March 2025, no evidence of tumor recurrence has been observed.
