# Supplementary figures and images for: Use of sintilimab in primary adenosquamous carcinoma of the liver results in pathological complete response: a case report and literature review
Source: Front Immunol. 2025 Apr 30;16:1578368. doi: 10.3389/fimmu.2025.1578368 (PMC12075158; doi:10.3389/fimmu.2025.1578368)

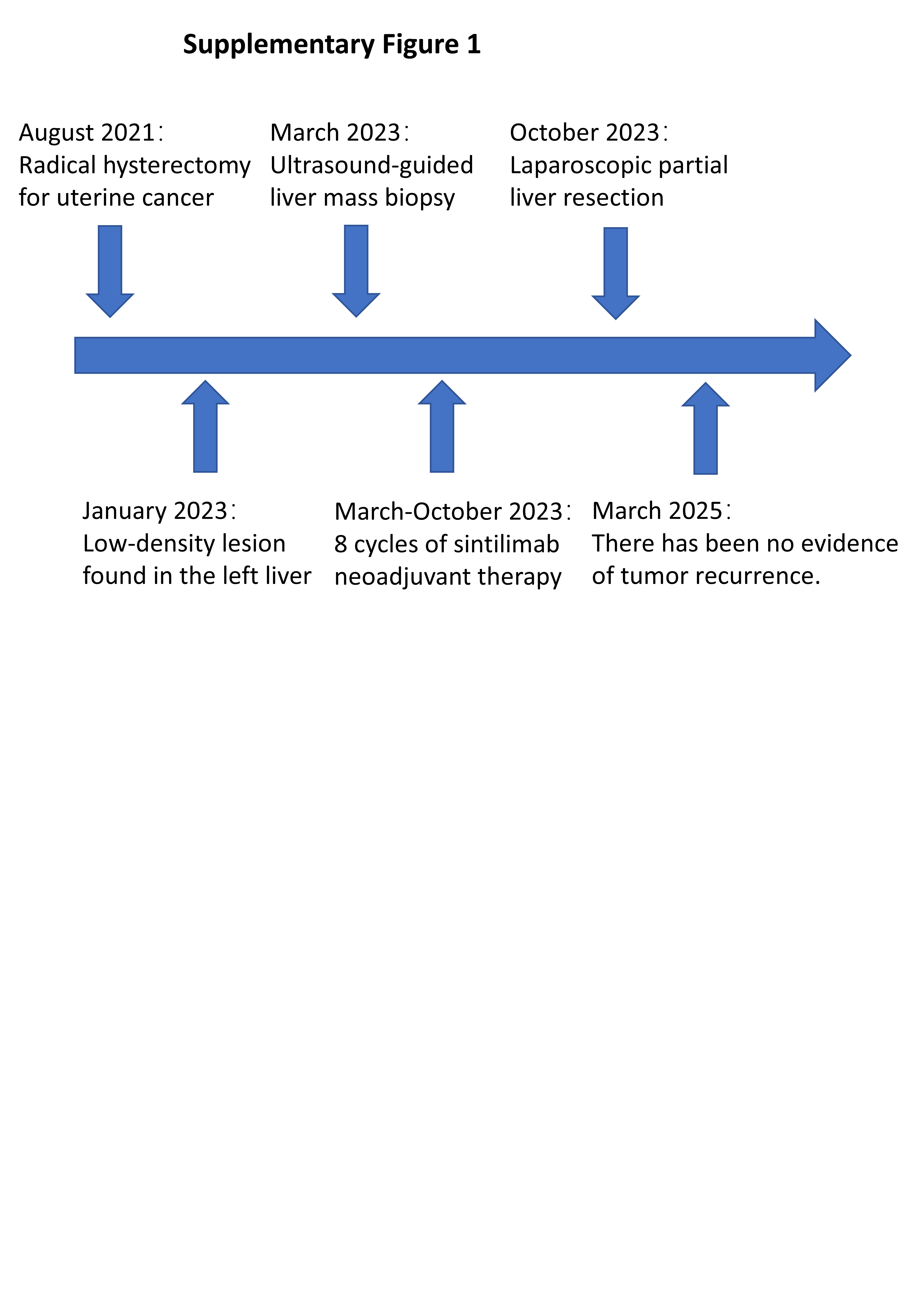

Supplement: Supplementary Figure 1 — Timeline of treatment and follow-up. Clinical timeline of diagnosis, treatment, and follow-up. The patient underwent a radical hysterectomy for uterine cancer in August 2021. In January 2023, a low-density liver lesion was detected during routine follow-up. A liver mass biopsy in March 2023 confirmed adenosquamous carcinoma (ASC) of the liver. The patient received eight cycles of sintilimab as neoadjuvant immunotherapy from March to October 2023, followed by laparoscopic partial liver resection. As of March 2025, no evidence of tumor recurrence has been observed. [file Image1.tif]
